# Differential Effect of TLR2 and TLR4 on the Immune Response after Immunization with a Vaccine against *Neisseria meningitidis* or *Bordetella pertussis*

**DOI:** 10.1371/journal.pone.0015692

**Published:** 2010-12-23

**Authors:** Floris Fransen, Rachel M. Stenger, Martien C. M. Poelen, Harry H. van Dijken, Betsy Kuipers, Claire J. P. Boog, Jos P. M. van Putten, Cécile A. C. M. van Els, Peter van der Ley

**Affiliations:** 1 Laboratory of Vaccine Research, Netherlands Vaccine Institute, Bilthoven, The Netherlands; 2 Department of Immunology and Infectious Diseases, Utrecht University, Utrecht, The Netherlands; Centre de Recherche Public de la Santé, Luxembourg

## Abstract

*Neisseria meningitidis* and *Bordetella pertussis* are Gram-negative bacterial pathogens that can cause serious diseases in humans. *N. meningitidis* outer membrane vesicle (OMV) vaccines and whole cell pertussis vaccines have been successfully used in humans to control infections with these pathogens. The mechanisms behind their effectiveness are poorly defined. Here we investigated the role of Toll-like receptor (TLR) 2 and TLR4 in the induction of immune responses in mice after immunization with these vaccines. Innate and adaptive immune responses were compared between wild type mice and mice deficient in TLR2, TLR4, or TRIF. TRIF-deficient and TLR4-deficient mice showed impaired immunity after immunization. In contrast, immune responses were not lower in TLR2−/− mice but tended even to be higher after immunization. Together our data demonstrate that TLR4 activation contributes to the immunogenicity of the *N. meningitidis* OMV vaccine and the whole cell pertussis vaccine, but that TLR2 activation is not required.

## Introduction

The innate immune system senses microbes through a number of receptors present on innate immune cells that can recognize a wide variety of microbial structures [1]. This group of receptors is often referred to as pattern recognition receptors (PRRs). There are several classes of PRRs, including Toll-like receptors (TLRs), C-type lectin like receptors, RIG-I like receptors, and Nod-like receptors. The TLR family is the best characterized class to date. In humans, 10 different TLRs have been described and each TLR recognizes distinct microbial structures [2]. For example, lipopolysaccharide (LPS), a major component of Gram-negative bacteria, activates TLR4, lipoproteins and several other structures activate TLR2, unmethylated CpG DNA of bacteria and certain viruses activate TLR9, and viral dsRNA is recognized by TLR3 [2]. Four adaptor proteins mediate TLR signalling: MyD88, TRIF, MAL, and TRAM [3]. All TLRs signal through MyD88, except TLR3, which signals solely through TRIF. Moreover, TLR4 is the only TLR which utilizes both MyD88 and TRIF [4]. Activation of these proteins eventually leads to induction of pro-inflammatory cytokines and type I interferon, respectively. Activation of TLR7/8 and TLR9 also leads to the induction of type I interferon, but in a MyD88-dependent manner [1].

The primary function of TLRs is to detect pathogens and activate innate immune cells to clear the infection immediately. However, TLRs also play an important role in the initiation of adaptive immune responses [5], [6]. Dendritic cells (DCs) are thought to play a central role in linking innate and adaptive immunity after TLR triggering, because of their superior capacity to stimulate T cells [7]. Which TLR is activated determines what types of cytokines and other factors are produced by the DCs, which in turn dictates whether the CD4^+^ T cells differentiate into Th1, Th2, Th17, or Treg [1],[8]. Because TLR ligands can both initiate and direct adaptive immunity, they have great potential as adjuvants. However, the claim that TLR activation always plays an important role in the induction of an adaptive immune response after vaccination has been challenged recently [9].

Many of the currently licensed vaccines are live attenuated strains or contain elements of killed microbes [5]. These vaccines likely contain structures that are recognized by TLRs and contribute to the immunogenicity. This has indeed been demonstrated for a number of vaccines [5], [10]–[13], but for the majority of vaccines this information is still lacking. It is important to identify the pathways induced by these successful vaccines for the rational design of new vaccines and/or adjuvants. Moreover, the human population is genetically very diverse and some individuals might have deficiencies in the pathways that are induced by the vaccine, which could explain why some individuals respond poorly after vaccination [14]. We decided to study the role of TLRs in the immunogenicity of two LPS-containing vaccines against the Gram-negative bacterial pathogens *Neisseria meningitidis* and *Bordetella pertussis*.

*N. meningitidis* is a leading cause of meningitis and sepsis worldwide [15]. The bacterium can be divided into several serogroups based on its capsule. For most serogroups (A, C, Y, and W-135) capsular polysaccharide vaccines are available, but not for serogroup B, because its capsular polysaccharide is not immunogenic. An attractive alternative for serogroup B is an outer membrane vesicle (OMV) vaccine [16]. OMV vaccines have been shown to be effective in controlling epidemics in Cuba, Norway, and New Zealand, where one particular clone of *N. meningitidis* serogroup B was causing high rates of meningococcal disease [16]. *B. pertussis* is the causative agent of whooping cough in humans. To prevent this disease, whole cell pertussis vaccines have been used for many decades in developed countries and are still used today in developing countries. However, due to adverse effects the whole cell vaccine has now been replaced in developed countries with a safer subunit vaccine consisting of a few *B. pertussis* antigens [17], [18]. The *N. meningitidis* OMV vaccine and whole cell pertussis vaccine both contain LPS and lipoproteins, which activate TLR4 and TLR2 respectively [19]–[23]. Ligands of these TLRs have been shown to have adjuvant activity in numerous studies in mice [11], [24]–[27].

Here we investigated the role of TLR2 and TLR4 in the induction of immune responses in mice after immunization with a *N. meningitidis* OMV vaccine and a whole cell pertussis vaccine. Innate cytokine induction, T cell responses, and antibody production were compared between wild type mice and mice deficient in either TLR2, TLR4, or TRIF. Surprisingly, TLR2−/− mice were not compromised in any of the responses after immunization. In contrast, TRIF-deficient and TLR4-deficient mice showed impaired immunity after immunization. We conclude that TLR4 activation contributes to the immunogenicity of the *N. meningitidis* OMV vaccine and the whole cell pertussis vaccine, but that TLR2 activation is not required.

## Results

### TRIF-deficient and TLR4-deficient mice have reduced innate cytokines levels after immunization

As a *N. meningitidis* OMV vaccine we chose OMVs derived from serosubtype P1.5-1,2-2. This serosubtype is one of the prevalent variants in The Netherlands. Moreover, serosubtype P1.5-1,2-2 is among the more immunogenic serosubtypes in humans and mice [28]–[30]. As a whole cell pertussis vaccine we used a mixture of the vaccine strains 134 and 509. In humans these vaccines are administered with alum as the adjuvant [16]–[18]. However, we did not include alum to study more specifically the intrinsic adjuvant activity of the vaccines.

**Figure 1:**
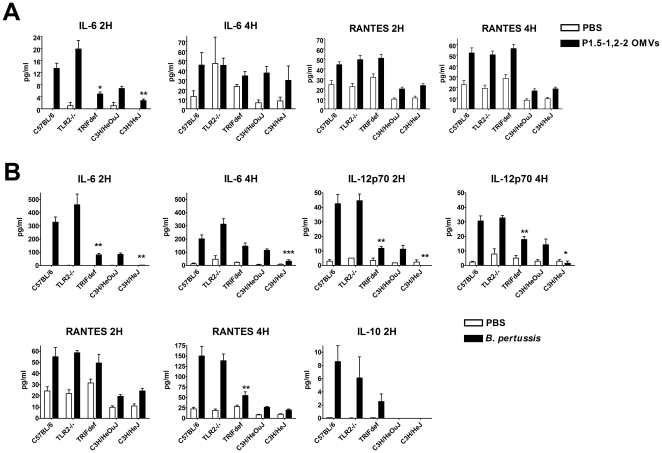
Serum cytokine levels shortly after vaccination. Two and four hours after immunization blood samples were taken from all mice and cytokine levels in the sera were analyzed with Luminex. Results for *N. meningitidis* OMVs are shown in panel A, results for whole cell pertussis vaccine are shown in panel B. There were 3 mice per group for the animals that received PBS and 6 mice per group for *N. meningitidis* OMVs and whole cell pertussis vaccine. The data are expressed as means, error bars represent S.E.M. An asterisk indicates a significant difference compared to the wild type group, * indicates p<0.05, ** indicates p<0.01, *** indicates p<0.001.

The role of TLR2 and TRIF in vaccine-induced responses after immunization was examined by immunizing wild type C57BL/6 mice, TLR2−/− mice, and TRIF-deficient mice, which all have a C57BL/6 background [31], [32]. To investigate the role of TLR4, responses were compared between TLR4-deficient C3H/HeJ mice and wild type C3H/HeOuJ mice [33]. All mouse strains were immunized subcutaneously with either PBS, *N. meningitidis* OMVs, or whole cell pertussis vaccine. After two and four hours a blood sample was taken from all mice to analyze serum cytokine levels of IL-1β, IL-6, IL-10, IL-12p70, RANTES, and TNF-α. In wild-type mice *N. meningitidis* OMVs induced modest levels of IL-6 and RANTES measurable at 2 and 4 h compared to PBS (Fig. 1A and data not shown). OMV-induced IL-6 levels at 2 hours were significantly lower in TRIF-deficient and TLR4-deficient mice and tended to be higher in TLR2−/− mice. Levels of OMV-induced RANTES were not impaired in the deficient mouse strains. Whole cell pertussis vaccine induced measurable levels of IL-6, IL-12p70, RANTES, and IL-10 (Fig. 1B and data not shown). Again TRIF deficient mice and TLR4-deficient mice were clearly compromised in cytokine induction compared to wild type mice. TLR2−/− mice were not impaired in cytokine production, IL-6 even tended to be higher. These results suggest that early serum cytokine production *in vivo* after immunization with *N. meningitidis* OMVs or whole cell pertussis vaccine requires mainly LPS signalling and not TLR2 activation.

### Comparison of antigen-specific antibody levels

**Figure 2:**
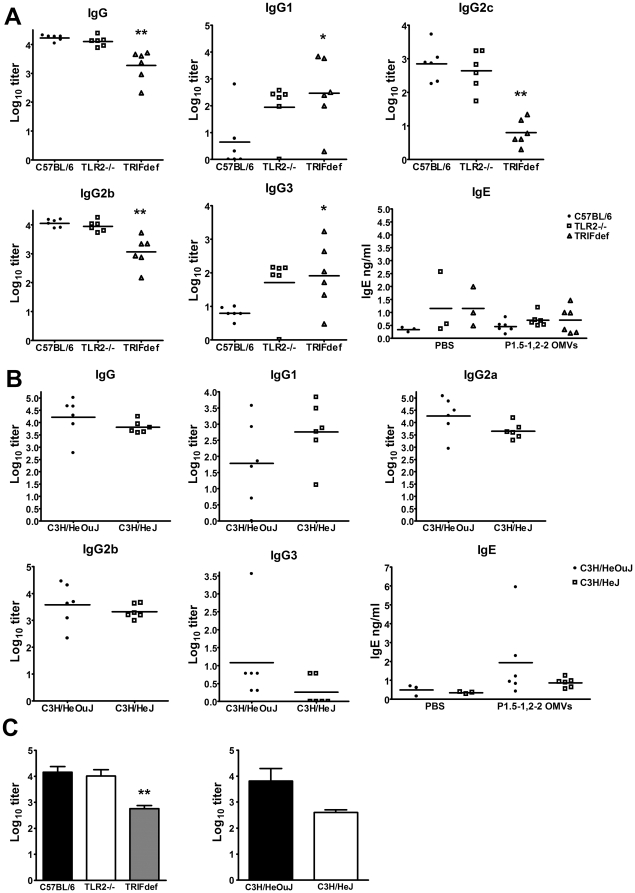
Antibody titers of mice after immunization with *N. meningitidis* P1.5-1,2-2 OMVs. Mice were immunized with *N. meningitidis* P1.5-1,2-2 OMVs and antigen-specific titers of IgG, IgG1, IgG2a/c, IgG2b, and IgG3 in sera were determined with ELISA. Also total IgE in sera of mice immunized with PBS or *N. meningitidis* P1.5-1,2-2 OMVs was measured with ELISA. Results for C57BL/6, TLR2−/−, and TRIF-deficient (TRIFdef) mice are shown in panel A, results for C3H/HeOuJ and C3H/HeJ mice are shown in panel B. Levels of serum bactericidal antibodies after immunization with *N. meningitidis* P1.5-1,2-2 OMVs are shown in panel C. Data are expressed as means of log_10_ titers for 6 mice per group, except levels of serum bactericidal antibodies in C57BL/6 and TLR2−/− mice (n = 12). Error bars represent S.E.M. An asterisk indicates a significant difference compared to the wild type group, * indicates p<0.05, ** indicates p<0.01.

The amount of antibodies was determined in all sera. It has been demonstrated previously that class switching to IgG2a/c and IgG2b depends on a Th1 response and class switching to IgG1 and IgE depends on a Th2 response [34]–[36]. Therefore, to analyze antibody production and the type of immune response that was induced, antigen-specific total IgG, antigen-specific IgG subclasses, and total IgE were determined in the serum. *N. meningitidis* OMVs induced a Th1-dependent antibody profile in wild type C57BL/6 mice, reflected by high levels of IgG2b and IgG2c, almost no IgG1, and no increase in total IgE compared to PBS injected mice (Fig. 2A). IgG3 levels were also very low. Strikingly, TLR2−/− mice immunized with *N. meningitidis* OMVs were not impaired at all in the antibody response, IgG1 and IgG3 levels even tended to be higher. On the other hand, *N. meningitidis* OMVs induced significant lower amounts of IgG in TRIF-deficient mice, which was mainly due to lower levels of IgG2b and IgG2c (Fig. 2A). In contrast, IgG1 and IgG3 levels to OMVs were significantly higher in TRIF-deficient mice compared to wild type mice. TLR4-deficiency did not significantly reduce antibody levels to OMVs, although for IgG, IgG2a, IgG2b, and IgG3 there was a tendency for reduced levels in TLR4-deficient mice (Fig. 2B).

Bactericidal antibodies are currently considered to be the most important correlate of protection for meningococcal disease [37], [38]. Furthermore, IgG2a/c, IgG2b, and IgG3, but not IgG1, isotypes can activate complement [39]. We tested the bactericidal activity of the sera of all mice immunized with *N. meningitidis* OMVs. Consistent with the lower IgG2b and IgG2c levels, TRIF-deficient mice had significantly lower levels of bactericidal antibodies compared to wild type mice (Fig. 2C). Similar amounts of bactericidal antibodies were induced in wild-type and TLR2−/− mice. Finally, TLR4 deficient mice tended to have less bactericidal antibodies than wild-type mice (Fig. 2D). Together these results suggest that LPS signalling also contributes to the generation of bactericidal antibodies after immunization with *N. meningitidis* OMVs, whereas TLR2 activation is not required.

**Figure 3:**
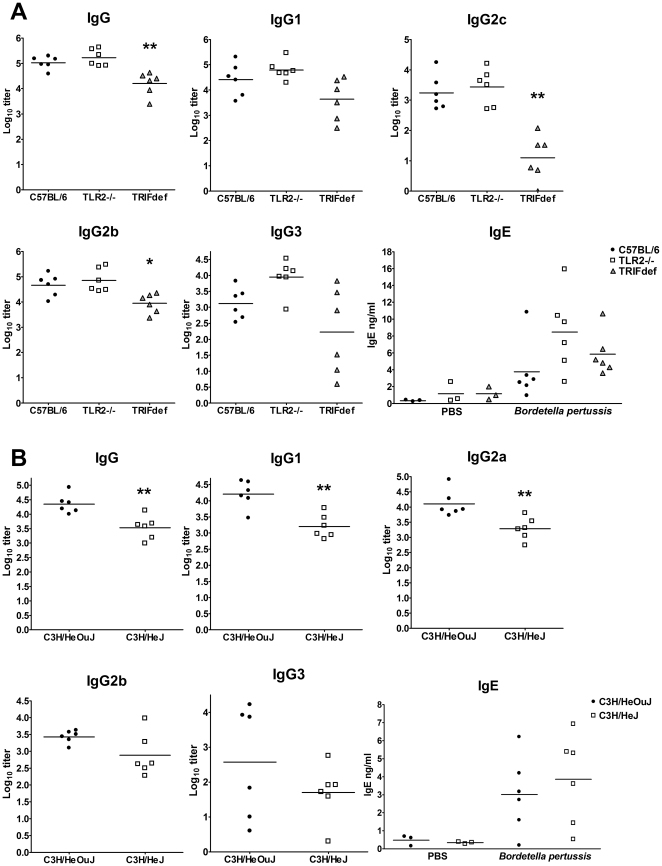
Antibody titers of mice after immunization with whole cell pertussis vaccine. Mice were immunized with whole cell pertussis vaccine (*Bordetella pertussis*) and antigen-specific titers of IgG, IgG1, IgG2a/c, IgG2b, and IgG3 in sera were determined with ELISA. Also total IgE in sera of mice immunized with PBS or whole cell pertussis vaccine was measured with ELISA. Results for C57BL/6, TLR2−/−, and TRIF-deficient (TRIFdef) mice are shown in panel A, results for C3H/HeOuJ and C3H/HeJ mice are shown in panel B. Data are expressed as means of log_10_ titers for 6 mice per group. An asterisk indicates a significant difference compared to the wild type group, * indicates p<0.05, ** indicates p<0.01.

**Figure 4:**
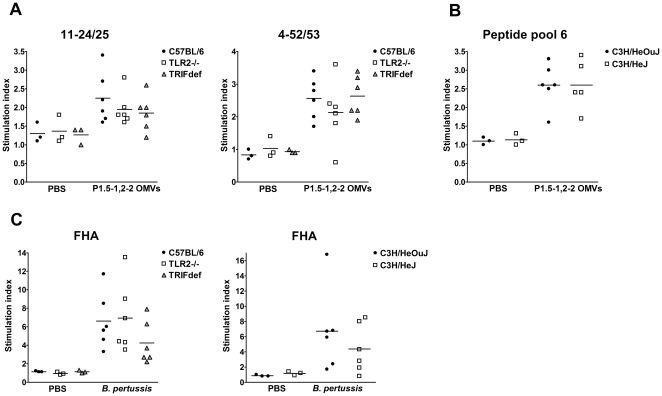
Proliferation of spleen cells after restimulation with antigen or peptide. Spleens were taken from the mice after immunizations and spleen cells were incubated for 4 days with medium, antigen, or peptides, after which [^3^H]thymidine incorporation was determined. Spleen cells of C57BL/6, TLR2−/−, and TRIF-deficient (TRIFdef) mice immunized with PBS (3 mice per group) or *N. meningitidis* P1.5-1,2-2 OMVs (6 mice per group) were restimulated with 1 µM of peptides 11–24/25 and 4–52/53 (A). Spleen cells of C3H/HeOuJ and C3H/HeJ mice immunized with PBS (3 mice per group) or *N. meningitidis* P1.5-1,2-2 OMVs (6 mice per group) were restimulated with peptide pool 6 (1 µM of each peptide, B). Spleen cells of C57BL/6, TLR2−/−, TRIF-deficient, C3H/HeOuJ, and C3H/HeJ mice immunized with PBS (3 mice per group) or whole cell pertussis vaccine (*B. pertussis*, 6 mice per group) were restimulated with 500 ng/ml of FHA antigen (C). Data are expressed as means of stimulation indices.

The differences between the different mouse strains in antibody levels were quite similar for the mice that received whole cell pertussis vaccine and the mice that received *N. meningitidis* OMVs. Again TLR2−/− mice did not show any defects in the induction of antibodies (Fig. 3A). Moreover, IgG3 and IgE levels tended to be higher in TLR2−/− mice compared to wild type mice. TRIF-deficient mice had significant lower amounts of IgG, which was mainly due to lower levels of isotypes IgG2b and IgG2c, as with *N. meningitidis* OMVs. However, in contrast to *N. meningitidis* OMVs, whole cell pertussis vaccine did not induce higher levels of IgG1 and IgG3 in TRIF-deficient mice. TLR4 deficient mice had lower amounts of all antibody isotypes compared to wild-type mice, except IgE (Fig. 4B). The differences were significant for IgG, IgG2a and IgG1. The whole cell pertussis vaccine also induced the production of total IgE, in contrast to *N. meningitidis* OMVs. In summary, our results demonstrate that LPS signalling contributes to the antibody response after immunization with *N. meningitidis* OMVs and whole cell pertussis vaccine. However, activation of TLR2 does not seem to contribute to the antibody response.

### Spleen cells of the different mouse strains proliferate equally after *in vitro* antigen restimulation

Also the spleen was taken from the mice immunized as described above. Spleen cells were restimulated with antigen or peptides for 4 days and proliferation of the cells was assessed by measuring [^3^H]thymidine incorporation. Spleen cells of mice with a C57BL/6 background and immunized with either *N. meningitidis* OMVs or PBS were restimulated with two PorA peptides, which were previously identified as two P1.5-1,2-2 PorA epitopes recognized by C57BL/6 mice (M. Poelen and C. van Els, unpublished data). Spleen cells from *N. meningitidis* OMV immunized mice clearly proliferated in response to the two peptides, suggesting that CD4^+^ T cells specific for those epitopes were generated after immunization (Fig. 4A). Interestingly, there was no difference between the three mouse strains in spleen cell proliferation. Which epitopes of P1.5-1,2-2 PorA were recognized by C3H/HeJ and C3H/HeOuJ mice was not known to us. Therefore, spleen cells of these mice immunized with either PBS or *N. meningitidis* OMVs were restimulated with different peptides pools spanning the entire P1.5-1,2-2 PorA protein sequence (data not shown). We found that one peptide pool induced the highest proliferation of spleen cells derived from *N. meningitidis* OMV immunized mice (Fig. 4B). There was also no difference in spleen cell proliferation between TLR4-deficient and wild-type mice. Together these results suggest that activation of TLR2 or TLR4 has no influence on CD4^+^ T cell proliferation after immunization with *N. meningitidis* OMVs.

To investigate CD4^+^ T cell proliferation after whole cell pertussis vaccine immunization, spleen cells of all the different mice immunized with whole cell pertussis vaccine or PBS were restimulated with a purified *B. pertussis* antigen, filamentous hemagglutinin (FHA, Fig. 4C). Clearly, only spleen cells of mice that received whole cell pertussis vaccine proliferated. Moreover, as with the PorA peptides, there were no significant differences between the mouse strains. Thus it seems that also for whole cell pertussis vaccine, TLR2 or TLR4 activation has little influence on CD4^+^ T cell proliferation.

### LPS signalling and TLR2 activation influence quality of T cell responses

**Figure 5:**
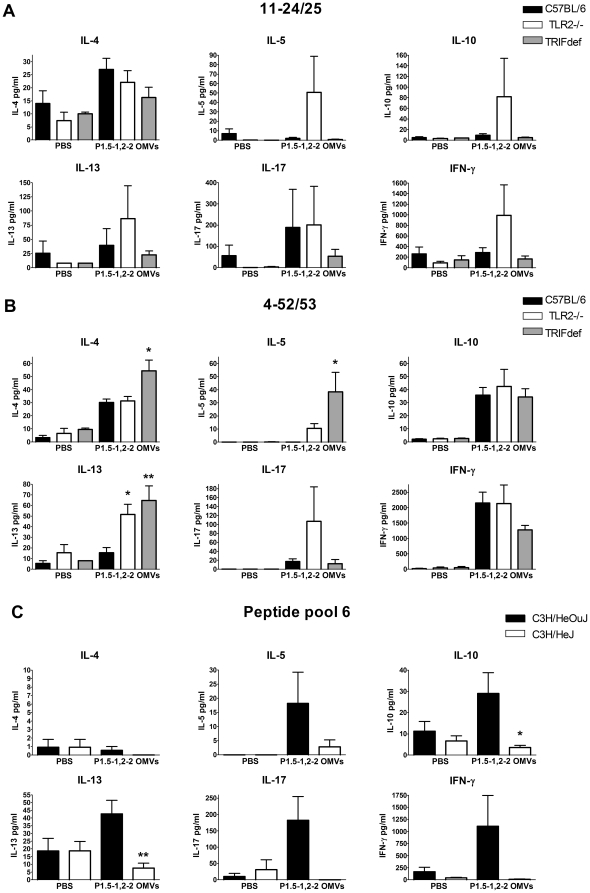
Cytokines produced by spleen cells after restimulation with P1.5-1,2-2 PorA peptides. Spleen cells from C57BL/6, TLR2−/−, or TRIF-deficient (TRIFdef) mice immunized with PBS or *N. meningitidis* P1.5-1,2-2 OMVs were incubated for 4 days with peptide 11–24/25 (A) or peptide 4–52/53 (B). Spleen cells from C3H/HeOuJ and C3H/HeJ mice immunized with PBS or *N. meningitidis* P1.5-1,2-2 OMVs were incubated for 4 days with peptide pool 6 (C). Cytokines IL-4, IL-5, IL-10, IL-13, IL-17, and IFN-γ were determined in the supernatant with Luminex. Data are expressed as means of three mice per group (PBS), or six mice per group (*N. meningitidis* OMVs), error bars represent S.E.M. An asterisk indicates a significant difference compared to the wild type group, * indicates p<0.05, ** indicates p<0.01.

Naïve CD4^+^ T cells can differentiate into Th1, Th2, Th17, or Treg cells. Th1 cells produce IFN-γ, Th2 cells produce IL-4, IL-5, and IL-13, Th17 cell produce IL-17, and most Treg cell subsets produce IL-10 [40], [41]. These cytokines were measured in the supernatant of the spleen cells stimulated with antigen or peptides to identify the type of T cell responses that were induced. Spleen cells of mice with a C57BL/6 background immunized with either *N. meningitidis* OMVs or PBS were restimulated with two P1.5-1,2-2 PorA peptides. Cytokines were analyzed in the supernatant for both peptides. Cells from TLR2−/− mice immunized with *N. meningitidis* OMVs tended to produce more IL-5, IL-10, IL-13, and IFN-γ after stimulation with peptide 11–24/25 (Fig. 5A). The other peptide, 4–52/53, generally induced higher production of cytokines by spleen cells of all the mice immunized with *N. meningitidis* OMVs (Fig. 5B). Moreover, peptide 4–52/53 induced spleen cells of TRIF-deficient mice to produce significant higher levels of IL-4, IL-5, and IL-13 and tended to produce less IFN-γ compared to spleen cells of wild type C57BL/6 mice. Clearly, the response was skewed towards a Th2 response in the TRIF-deficient mice, consistent with what we noticed at the antibody level (Fig. 2A). Spleen cells of TLR2−/− mice also produced significant higher levels of IL-13 than wild type spleen cells (Fig. 5B). Furthermore, IL-5 and IL-17 tended to be higher. Spleen cells of TLR4-defcient and wild-type mice were restimulated with different peptide pools. Since peptide pool 6 induced the highest proliferation, cytokine production by cells stimulated with this pool was measured (Fig. 5C). Remarkably, spleen cells from TLR4-deficient mice immunized with *N. meningitidis* OMVs did not produce any of the cytokines analyzed, although the cells clearly proliferated as much as the cells from wild-type mice (Fig. 4B). In contrast, wild-type cells produced all cytokines, except IL-4. Overall these results show that after immunization of wild type mice with *N. meningitidis* OMVs, antigen-specific T cells mainly produce IFN-γ. This suggests that *N. meningitidis* OMVs mainly induce a Th1 response in wild type mice. We also demonstrate that some antigen-specific T cells produce IL-17. Furthermore, our results suggest that TLR4 activation is required for the generation of cytokine producing T cells and that TRIF signalling contributes to a Th1 response. Finally, TLR2 activation is not required for the generation of cytokine producing T cells, but in contrast seems somewhat inhibitory.

**Figure 6:**
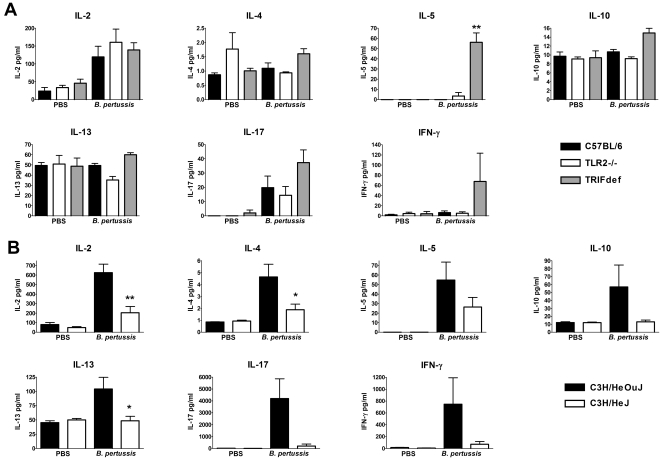
Cytokines produced by spleen cells after restimulation with FHA. Spleen cells from C57BL/6, TLR2−/−, TRIF-deficient (TRIFdef) mice (A), or C3H/HeOuJ and C3H/HeJ mice (B) immunized with PBS or whole cell pertussis vaccine were incubated for 4 days with 500 ng/ml FHA Cytokines IL-2, IL-4, IL-5, IL-10, IL-13, IL-17, and IFN-γ were determined in the supernatant with Luminex. Data are expressed as means of three mice per group (PBS), or six mice per group (whole cell pertussis vaccine), error bars represent S.E.M. An asterisk indicates a significant difference compared to the wild type group, * indicates p<0.05, ** indicates p<0.01.

Spleen cells of the different mouse strains immunized with whole cell pertussis vaccine were restimulated with purified FHA antigen. The strong proliferation of cells of mice with a C57BL/6 background after antigen restimulation was associated with the presence of IL-2 and IL-17 (Fig. 6A). In addition, low amounts of IFN-γ and IL-5 were only produced by the spleen cells of TRIF-deficient mice immunized with whole cell pertussis vaccine. FHA stimulated spleen cells of wild-type C3H/HeOuJ mice immunized with whole cell pertussis vaccine produced all cytokines measured (Fig. 6B). Levels of IL-17 were especially high followed by IFN-γ. In contrast, reminiscent of the *N. meningitidis* OMVs model, FHA stimulated spleen cells of TLR4-deficient C3H/HeJ mice hardly produced any cytokines, even though they proliferated as much as wild-type C3H/HeOuJ spleen cells. Together, our results demonstrate that whole cell pertussis vaccine immunization induced a Th17/Th1 response, which was impaired in TLR4-deficient mice.

## Discussion

In the present study, we demonstrate that TLR4 signalling by LPS contributes to generation of adaptive immune responses after immunization with *N. meningitidis* OMVs or whole cell pertussis vaccine, while TLR2 activation was not required. Instead, TLR2−/− mice overall showed higher rather than lower responses compared to C57BL/6 mice after immunization with the *N. meningitidis* and *B. pertussis* vaccines. For example, IL-6 serum levels just after vaccination tended to be higher in the

TLR2−/− mice. At the antibody level, IgG1 and IgG3 tended to be higher in *N. meningitidis* OMVs immunized TLR2−/− mice, whereas IgG3 and IgE tended to be higher in TLR2−/− mice immunized with whole cell pertussis vaccine. Finally, spleen cells of TLR2−/− mice immunized with *N. meningitidis* OMVs restimulated with P1.5-1,2-2 PorA peptides produced higher levels of several cytokines, including significantly higher levels of IL-13. Together these results indicate that TLR2 activation inhibited immune responses, especially Th2 responses, after vaccination. Similarly, TLR2-deficient mice were shown to have greatly enhanced Th1 and Tc1 responses after vaccination with yellow fever vaccine YF-17D [13]. Moreover, it has been demonstrated recently that activation of TLR2 by the yeast cell wall derivative zymosan induces a Treg response [42], [43]. On the other hand, TLR2 ligands have been shown to have adjuvant activity in several studies [10], [11], [27], [44]. In addition to induction of a Treg response, TLR2 activation has also been shown to induce a Th1 reponse [45], or Th2 response [46]–[48]. How TLR2 activation can lead to all these different responses is currently not clear, but it might depend for example on which ligand is used, whether the ligand is recognized by TLR2/TLR1 or TLR2/TLR6, or which cell type is targeted. In any case, our results show that TLR2 activation is not required for induction of adaptive immunity when TLR4 is also activated. However, even TLR4-deficient mice still develop adaptive immune responses after vaccination. This residual response might be due to TLR2 activation. To address this issue, also mice deficient in both receptors should be tested. Moreover, the vaccines might contain ligands of other TLRs or PRRs, which also contribute to the immunogenicity.

TRIF-deficient mice clearly showed altered immune responses after immunization with *N. meningitidis* OMVs and whole cell pertussis vaccine. Antigen- specific IgG2b and IgG2c levels were significantly reduced for both vaccines, which resulted in less bactericidal antibodies in the case of *N. meningitidis* OMVs. In addition, spleen cells of TRIF-deficient mice immunized with *N. meningitidis* OMVs restimulated with PorA peptides produced significantly higher amounts of IL-4, IL-5, and IL-13, while IFN-γ production was reduced. Together these data show that activation of the TRIF pathway leads to a Th1 response after immunization with *N. meningitidis* OMVs or whole cell pertussis vaccine. Therefore, targeting of the TRIF pathway is important, because for both vaccines optimal responses after vaccination should include Th1-responses. Induction of the TRIF pathway eventually leads to the induction of type I interferon, which is not induced by the MyD88 pathway downstream of TLR2 or TLR4 [3], [32], [49]. We have shown previously that ligands of TLR3, TLR4, TLR7, and TLR9 enhance the immunogenicity of a *N. meningitidis* outer membrane vaccine by promoting Th1-dependent antibody production, whereas ligands of other TLRs did not show adjuvant activity [25]. Importantly, activation of TLR3, TLR4, TLR7, and TLR9 leads to the induction of type I interferon, but activation of the other TLRs does not [50]. Possibly, TLR-induced type I interferon drives Th1 development and contributes to the adjuvant effect of TLR ligands. Indeed, the adjuvant effect of poly IC by inducing CD4^+^ Th1 immunity and humoral immunity depends on type I interferon [51], [52]. Interestingly, the vaccine adjuvant Monophosphoryl lipid A, a low-toxicity LPS derivative, has been shown to preferentially activate the TRIF pathway [53].

We also show that TLR4-deficient mice have impaired immune responses after immunization with *N. meningitidis* OMVs and whole cell pertussis vaccine. After whole cell pertussis immunization of the TLR4-deficient C3H/HeJ mice, IL-6 and IL-12p70 were almost undetectable in the serum. Moreover, IgG, IgG1, and IgG2a levels were significantly lower in these mice. In contrast, others have found that antibody levels are not decreased in TLR4-deficient mice after immunization with whole cell pertussis vaccine [54], [55]. However, in these studies alum was added to the vaccine as an adjuvant, which mainly enhances humoral immunity. Presumably, the adjuvant effect of LPS is more redundant in the presence of another potent adjuvant like alum.

Surprisingly, TLR4-deficient C3H/HeJ mice did not have significantly decreased OMV-directed antibody titers compared to wild-type C3H/HeOuJ mice. By contrast, we previously found that LPS-deficient *N. meningitidis* outer membrane complexes (OMCs) induced more than 1000-fold lower antibody titers in TLR4-proficient mice than wild type OMCs [25]. This suggests that LPS improves the immune response not only by activating TLR4, but also by other mechanisms. Possibly, LPS could target the vaccine to antigen presenting cells by binding to other receptors, for example CD14 or C-type lectins.

In contrast to antibody levels, cytokine production after restimulation with PorA peptides was severely impaired in spleen cells from TLR4-deficient C3H/HeJ mice. Similarly, it has been demonstrated that whole cell pertussis vaccine immunization led to comparable antibody titers in TLR4-deficient and wild type mice, but IFN-γ and IL-17 production of T cells after antigen restimulation was much lower for TLR4-deficient mice [55]. These results suggest that TLR4 activation is more important for the induction of effector T cells than for antibody production. Remarkably, the TLR4-deficient spleen cells restimulated with PorA peptides or FHA proliferated equally well as spleen cells from the wild-type mice. This suggests that in TLR4-deficient mice, antigen-specific CD4^+^ T cells were generated without effector function. Interestingly, it has been shown that DCs that had a mature phenotype but did not produce IL-12 and other pro-inflammatory cytokines, promoted expansion of CD4+ T cell populations lacking effector function [56]. Possibly, this type of DCs was induced in the TLR4-deficient mice. After whole cell pertussis vaccine immunization, serum levels of IL-6 and IL-12p70 were very low in C3H/HeJ mice. In addition, it has been demonstrated that serum IL-12p70 production in vivo after administration of a TLR9 ligand depended entirely on DCs [57]. Unfortunately, in our experiments *N. meningitidis* OMVs were not potent enough to enhance serum IL-12p70 levels even in wild-type animals, but in a recent report where a 10-fold higher dose of a comparable preparation of native *N. meningitidis* OMVs was used, IL-12p70 was elevated in serum of wild-type mice but not in TLR4-deficient mice. Serum levels of MCP-1, TNF-α, IL-6, and IL-10 were also much lower in the TLR4-deficient mice [58].

In conclusion, our results show that two successful LPS-containing vaccines against the Gram-negative bacterial pathogens *N. meningitidis* and *B. pertussis* require TLR4 signaling for optimal immunogenicity and induction of Th1 responses in mice. On the other hand, TLR2 activation was not required. These findings confirm that TLR4 and the TRIF pathway are attractive targets for adjuvants in vaccines.

## Materials and Methods

### Ethics statement

Animal experiments were approved by the Netherlands Vaccine Institute's Committee on Animal Experiments (DEC), project review nrs. 200800223 and 200800224.

### Animals

Specific-pathogen-free C57BL/6J, C3H/HeOuJ (TLR4-proficient) and C3H/HeJ (TLR4-deficient) mice were purchased from Charles River Laboratories. TLR2−/− mice [31] and TRIF-deficient mice [32], both from a C57BL/6 background, were kindly provided respectively by Dr. Shizuo Akira (Osaka University, Japan) and Dr. Bruce Beutler (Scripps Research Institute, La Jolla, CA) via Dr. Tom van der Poll (Academic Medical Center, Amsterdam, the Netherlands). For experiments, female mice were used, aged 10–14 weeks.

### Vaccine preparation

The OMV vaccine was obtained from the PorB/RmpM-negative *N. meningitidis* strain TR52 [P1.5-1,2-2] [59] by extraction of bacteria with 0.5% deoxycholate in 0.1 M Tris-HCl-10 mM EDTA (pH 8.6) and were purified by differential centrifugation [60]. OMVs were stored at 4°C and diluted prior to immunization to 20 µg/ml in PBS. For preparation of the whole cell pertussis vaccine, *B. pertussis* strains 134 and 509 were grown in defined synthetic medium [61]. After 18–22 hours of culture, bacteria were heat inactivated for 10 min at 56°C in the presence of 16 mM formaldehyde. Next cells were centrifuged for 10 min at 16,100×g and resuspended in PBS to 200 international opacity units (IOU)/ml. The suspensions were stored at 4°C. Prior to immunization, both strains were mixed 1∶1 and diluted to a final concentration of 16 IOU/ml in PBS.

### Immunizations

Mice were immunized subcutaneously on days 0 and 21 with 250 µl PBS or 5 µg of *N. meningitidis* P1.5-1,2-2 OMVs in 250 µl PBS. Whole cell pertussis vaccine (4 IOU in 250 µl PBS) was administered subcutaneously on days 0 and 28. Groups that received PBS contained 3 mice per group and groups that received OMVs or whole cell pertussis vaccine contained 6 animals per group. A blood sample of all mice was taken 2 and 4 hours after immunization. From the mice that received PBS or *N. meningitidis* P1.5-1,2-2 OMVs blood and spleen were taken on day 28. From the mice that received whole cell pertussis vaccine blood and spleen were taken on day 42. Sera were collected and stored at −20°C. Single-cell suspensions of spleen cells were produced by mechanical dissociation of organs through 70-µm-pore-size nylon filters. Spleen cells were used either freshly or after freezing and storage at −135°C.

### Antigen-specific antibodies

Antigen-specific IgG, IgG1, IgG2a/c, IgG2b, and IgG3 antibodies were determined by enzyme-linked immunosorbent assay (ELISA) as described previously [25] with slight modifications. Briefly, flat-bottom 96-well microtiter plates (Immulon 2; Nunc) were coated overnight at room temperature with 100 µl/well 5 µg/ml *N. meningitidis* P1.5-1,2-2 OMVs (4 µg/ml PorA) in PBS, or 2 IOU/ml of *B. pertussis* strain 509 (inactivated as described above) in PBS. Plates were incubated with serial dilutions of the sera of the immunized mice (100 µl/well) at 37°C for 1 h. Antibody isotypes were detected with anti-mouse antibodies conjugated to horseradish peroxidase (Southern Biotechnology Associates, Inc.). Next, peroxidase substrate was added to the wells and the reaction was stopped by the addition of 2 M H_2_SO_4_. A four-parameter curve fit was made for optical density values of the serial dilutions, and the antibody titer was calculated in reciprocal dilutions that gave 50% of the maximum absorbance. The results are expressed as log_10_ titers.

### Total IgE

Total IgE levels in the sera were measured by ELISA as described previously [25]. Briefly, flat-bottom 96-well microtiter plates were coated with rat anti-mouse IgE monoclonal antibody (BD Biosciences) and incubated overnight at 4°C. Next, the plates were incubated for 1 h at room temperature with the sera of the immunized mice and purified mouse IgE clone C48-2 (BD Biosciences) as a standard. For detection of IgE, biotinylated anti-mouse IgE (BD Biosciences) was used followed by streptavidin-HRP (Sanquin). Peroxidase substrate was used as a substrate, and the reaction was stopped with 2 M H_2_SO_4_. The absorbance was determined at 450 nm.

### Serum bactericidal assay

The sera of immunized mice were diluted 1∶5 in Gey's balanced salt solution plus 0.5% bovine serum albumin and then heat inactivated for 30 min at 56°C. Next, twofold serial dilutions of the sera, together with *N. meningitidis* P1.5-1,2-2 (10^4^ CFU/ml), were incubated in 96-well plates at room temperature for 15 min. After the addition of baby rabbit complement (20% of total volume) plates were incubated at 37°C for 1 h. Bacteria were plated on GC medium base (Difco Laboratories) supplemented with IsoVitaleX (Becton Dickinson) and grown overnight at 37°C in 5% CO_2_ in a humid atmosphere. The serum bactericidal titer was determined as the reciprocal serum dilution that gave more than 90% killing of the number of bacteria used. The results are expressed as log_10_ titers.

### Peptide synthesis

Overlapping 18-mer peptides covering the entire *B. pertussis* P.69 Prn protein or *N. meningitidis* P1.5-1,2-2 PorA protein were prepared by solid-phase synthesis using *N^α^*-(9-fluorenyl)methoxycarbonyl (FMOC)-protected amino acids and a Syro II simultaneous multiple-peptide synthesizer (MultiSyntech GmbH, Witten, Germany). The purity and identity of the synthesized peptides were assessed by reverse-phase high-performance liquid chromatography and mass spectometry.

### Spleen cell proliferation

Spleen cells from immunized mice were cultured at 1.5×10^5^ in 150 µl IMDM (Gibco BRL) supplemented with 100 units/ml penicillin, 100 µg/ml streptomycin, 300 µg/ml L-glutamine (Gibco BRL), 10% heat-inactivated fetal calf serum (FCS) (Gibco BRL), and 50 µM beta-mercaptoethanol in 96-well round-bottom plates (Greiner). Spleen cells were co-cultured with either medium, 1 µM of P1.5-1,2-2 PorA peptides, or 500 ng/ml purified FHA (Kaketsuken). On day 4, 100 µl of supernatant was removed and stored at −20°C. Next, 0.5 µCi (18.5 kBq) [^3^H]thymidine (Amersham) was added to the wells, and cells were cultured for another 18 h. Cells were harvested, and [^3^H]thymidine incorporation was determined as counts per minute using a Wallac 1205 Betaplate liquid scintillation counter. Results are expressed as stimulation indices from triplicate wells, calculated as (counts per minute of cultures in the presence of antigen)/(counts per minute of cultures in the presence of medium only).

### Luminex

A 6-plex Bio-Plex assay (Bio-Rad) containing beads for mouse IL-1β, IL-6, IL-10, IL-12p70, RANTES, and TNF-α was used to measure levels of these cytokines in the sera of the mice taken 2 and 4 hours after immunization. A 7-plex Bio-Plex assay (Bio-Rad) containing beads for mouse IL-2, IL-4, IL-5, IL-10, IL-13, IL-17, and IFN-γ was used to determine amounts of these cytokines in the supernatants of spleen cells restimulated with antigen or peptide. Cytokine concentrations were determined with a Bio-Plex system (Bio-Rad).

### Statistics

One-way analysis of variance (ANOVA) was performed, followed by the post-hoc Dunnett *t* test, to analyze differences in means between the experimental groups of C57BL/6 mice, TLR2−/−, and TRIF-deficient mice (GraphPad Prism 4). The unpaired two-tailed Students' t test was used to analyze differences in means between the experimental groups of C3H/HeOuJ and C3H/HeJ mice. Variances were compared with the F-test. In case of significantly different variances (p<0.05), the Welch's correction was included (GraphPad Prism 4). In case the SD of one of the means was 0, the One-sample T test was used instead (SPSS Statistics 17.0). P values of <0.05 were considered significant.
